# Design and implementation of a health systems science curriculum at a large teaching hospital

**DOI:** 10.1186/s12909-022-03706-y

**Published:** 2022-08-25

**Authors:** Elizabeth Harcher, Adeola Fakolade, Dana Gordon, Susan Nedorost

**Affiliations:** University Hospitals Graduate Medical Education, Lakeside 6223, 11100 Euclid Ave, Cleveland, Ohio (OH) 44106 USA

**Keywords:** Health systems science education; Population health education; Graduate medical education competencies

## Abstract

**Background:**

Physicians must increasingly lead change for improvement in the value of health care for individuals and populations. Leadership, stewardship, and population health competencies are not explicitly part of the Accreditation Council for Graduate Medical Education (ACGME) requirements and are best appreciated in the context of Health Systems Science (HSS).

HSS education is best approached at the institutional level, yet almost all graduate medical education (GME) curriculum is at the program level. We describe the process of designing and implementing an institutional HSS GME curriculum in a hospital-based sponsoring institution.

**Methods:**

A group of diverse stakeholders drafted a curriculum to build competencies in leadership, stewardship, and population health, which was further refined by our Graduate Medical Education Committee (GMEC) and Resident Forum in the academic years 2015–2017. The refined curriculum was implemented at the institutional level of a large urban teaching hospital with over 80 ACGME accredited programs in the 2017–2018 academic year, participation was tracked and impact surveys were conducted.

**Results:**

All programs participate in at least parts of the curriculum with sustained use. Annual surveys show a progression in assessment of our target competencies and/or opportunities to reflect and provide feedback. The annual program review meeting and GMEC meetings are used to troubleshoot and identify new curricular opportunities.

**Conclusion:**

This innovative institutional curriculum has been sustained for over four years and we believe that other training institutions with similar goals will find our experience implementing an institutional curriculum translatable to their clinical learning environment.

**Supplementary Information:**

The online version contains supplementary material available at 10.1186/s12909-022-03706-y.

## Background

Future physicians will need competencies beyond those currently measured by the Accreditation Council for Graduate Medical Education (ACGME) milestones [1]. Physicians must graduate from an ACGME accredited training program to become eligible to sit for American Board of Medical Specialties (ABMS) board examinations in the United States. ABMS board certification consists of assessment of medical knowledge, and continuing certification requires quality improvement efforts. While the ACGME has outlined the expected transformation of GME to meet the needs of the populations we serve [2], there is a national need for changes in graduate medical education to develop new competencies to meet the quadruple aim. The articulation of the quadruple aim documents that our current health care system has gaps in team leadership of patient and provider experience, value of care, and population health [3].

The current competencies largely focus on the role of the physician in individual patient care, while the additional competencies focus on the role of the physician within an integrated health care delivery system focused on population health. Physicians will require leadership competencies to implement these changes and will increasingly be expected to collaborate with advanced practice providers and other members of the health care team, functioning as master clinicians and leading process improvement [2].

Internal Medicine was the first discipline to consider additional competencies that future physicians will need [4] and others medical specialties followed. Physician competencies needed in the future include expert judgement and a high degree of professionalism and ethics to manage complex issues that exceed the capability of artificial intelligence and an algorithmic approach to care [3]. Teams will include those with expertise beyond medicine to enhance population health [2]. We view the skills of change management, ethical practice, and teamwork as leadership competencies. “Increasing economic pressures will intensify the demand for high value care for individuals and populations” [3]. We view quality improvement and value- based care as stewardship competencies. We consider understanding social determinants of health and data analysist (including looking for disparities in data sets) to be population health competencies. Consolidating competencies into Leadership, Stewardship, and Population Health simplified communication regarding our goals.

Table 1 compares currently measured ACGME competencies and additional competencies we believe are needed for physicians to fully contribute to health care reform. Guided by the quadruple aim, we distilled a number of competencies to summarize those we felt were most critical to manage change from volume to value of care, while involving other professionals as part of the care team to mitigate burden on the individual physician.

**Table 1:** Legend: Competencies currently measured in graduate medical education and additional competencies needed

Now	Additional
Medical Knowledge	Population health/ preventive medicine
Patient Care	Collaborative Leadership
Interpersonal and Communication Skills	Team dynamics and change management
Practice Based Learning and Improvement	Population and patient data
Systems Based Practice	Cost conscious care
Professionalism	Personal and team well-being

These additional competencies are best appreciated in the context of health systems science (HSS) [5]. HSS is now considered the “third pillar” of medical education along with basic and clinical science. HSS is most effectively demonstrated in an inter-disciplinary learning environment, yet most graduate medical education (GME) occurs at the program level within a single discipline.

## Methods

Our GME leaders established meetings and worked collaboratively with senior institutional leaders, medical educators, and trainees, to create an experiential HSS curriculum utilized by all GME training programs in our large teaching hospital. We wanted to integrate this curriculum seamlessly with our health system’s clinical transformation and quality and safety initiatives to improve value of care for patients (stewardship) and for communities we serve (population health). Our desire is to empower our graduates to utilize their acquired knowledge, skills, and attitudes not only during training but throughout their career. This requires intrinsic motivation gained by satisfaction with learning experiences in a supportive environment [6].

Our overarching goal was to create an institutional HSS curriculum. Our curricular goals were:

1. Create a unified set of experiences shared across disciplines to build competencies in leadership, stewardship, and population health.
2. Build upon existing undergraduate curricula in Health Systems Science (HSS) that have recently evolved.
3. Provide our graduates with the relevant knowledge, skills, and attitudes that impact their future practice.

### Context

We are a large urban teaching hospital sponsoring over 80 ACGME accredited programs enrolling over 800 residents and fellows. We wanted to ensure incorporation of this training in every program and in an inter-disciplinary fashion whenever possible. We believe that curriculum transcending disciplines is very important for Health Systems Science as we train future physicians who will contribute to team and population well-being, as well as to the health of individual patients. Aside from onboarding orientation modules, we know of few other large institutions that have an institutional curriculum. A suggested framework for Health Systems Science Education was recently published [7]. We describe the methods for design and implementation of our HSS curriculum.

We began the initiative in 2015. Many stakeholders (approximately 60) took part in review and discussion of the curriculum during the subsequent year. A GME Advisory task force comprised of program directors (some of whom are Graduate Medical Education Committee [GMEC] members) and department chairs met to discuss the HSS curriculum monthly during the 2016–2017 academic year. Our Resident Forum, comprised of peer-selected residents from each training program, and the GMEC, comprised of program directors from a variety of disciplines, also included this as an agenda item during regularly scheduled monthly meetings. Hospital leaders, including the Chief Operating Officer and affiliated medical school leaders including the Assistant Dean for Health Systems Science and the Vice Dean for Education, also offered guidance in individual meetings.

We chose Leadership, Stewardship, and Population Health to categorize our target competencies. Leadership includes professionalism, patient-centered communication, and teamwork. Stewardship includes quality improvement for high value care. Population health includes stewardship of quality improvement projects and stewardship for groups of patients. Once the desired competencies were articulated, we began to design curriculum.

The first draft of the curriculum was detailed and included some course work similar to a Master’s in Public Health degree. We iteratively simplified the curriculum and made it less didactic and more experiential. We included perspectives on feasibility from multiple programs including medical, procedural, and hospital-based disciplines. Consensus was reached after one year, when no further changes were proposed.

### Learning objectives and curriculum

The curriculum is divided into 3 competencies, with learning opportunities for program directors/faculty and residents/fellows. Table 2 is a summary of the main activities.

**Table 2:** Summary of main components of the health systems science curriculum

Competency	Activity for Program Directors/Faculty	Activity for Residents/Fellows
Leadership	One leadership course at hospital or affiliated university	One on-line course (e.g. unconscious bias training; innovation by design) AND One reflective essay on patient centered communication
Stewardship	Use a new patient decision aid OR Discuss a Choosing Wisely topic at a faculty meeting	Have a patient attend one didactic session each year to explain the value of each encounter in their disease journey AND Complete coding and documentation training
Population Health	Look for disparities in a health data set and involve a trainee OR Participate in a registry and demonstrate to a trainee OR Read a book about population health and send a brief report to GMEC for inclusion in our list	Participate in the See the City You Serve Orientation tour (only required once during training) AND View a Patient Safety/QI module or attend a live session where safety reports are vetted AND Write a reflective essay on a systems based issue

### Assessment methods

Our primary goal was to create an institutional curriculum. The institutional, rather than program level, implementation, helped underscore the importance of our work. Obtaining buy-in and participation from over 80 different programs was the primary success metric.

The annual internal GME faculty and resident surveys were modified in the 2016–2017 (baseline) academic year to include questions about frequency of assessment or personal reflection on competencies included in the curriculum. These questions were subsequently included in each annual survey after the HSS curriculum was implemented in 2017–2018 to assess trends in utilization and impact on clinical work. The results are discussed in the next section and the data is in supplementary tables.

## Results

### Goal #1: Create a unified set of experiences shared across disciplines to build competencies in leadership, stewardship, and population health

We included resources from our health system Leadership Institute, Quality Institute and GME office to encourage experiential learning in the areas of desired competencies. All residents are required to participate each year, and program directors participate to role model lifelong learning.

Learning activities are differentiated for program directors/ faculty and for residents/fellows. Program director participation is mandatory, but faculty participation is optional at the discretion of the program. The program director and faculty expectations are set to promote role-modeling of life-long learning over an interval of many years, while the resident/fellow expectations are set to acquire competencies in the shorter interval of training.

Learning objectives for Leadership include incorporating reflection in self-improvement of communication. We allowed program directors flexibility in implementation for either individual or group reflection. The online curriculum includes a link to an Association of Professors of Dermatology resource with suggested topics for reflection on communication in the clinical teaching environment. This link also includes a scoring rubric for assessing written essays that may be utilized by the Clinical Competency Committee (CCC) charged with assessing communication skills in ACGME accredited programs. We suggest that residents and fellows frame reflections in a way that will make them useful as preparation for future job or fellowship interviews when asked to respond to behavioral questions. For example, residents might write about caring for a patient whose values conflicted with their own and discuss this as a growth experience.

Reflective essays induced anxiety, and the Graduate Medical Education Committee had concerns about requiring reflective essays in terms of evidence for educational value, time required for program leaders to read the essays, and risk of discovery in the event of a medical malpractice claim. We addressed these by providing literature demonstrating benefit [8]. We also asked program directors to consider the reflective essays to be direct observation tools for their Clinical Competency Committees to use for assessing milestones in Interpersonal Communication, Professionalism, and System Based Practice, although a single writing sample should never be used alone for a high stakes assessment [9]. Our institutional legal counsel suggests that all providers consider any written document whether personal or professional to be potentially discoverable, and we reminded program directors to share this with incoming trainees. Some programs substituted group reflective discussion for the essays.

Learning objectives for Stewardship included knowledge of resources such as Choosing Wisely and experience with understanding patient perspective of value in their care. All programs are required to have a patient participate in one didactic session each year where the patient can explain their perception of value in each step of their disease journey. This is intended to develop the skill of listening for value from a patient perspective.

Learning objectives for Population Health included knowledge of the populations we serve, specifically available local resources and challenges. Presented at institutional orientation as a bus (or virtual in the pandemic) tour of inner ring neighborhoods near our flagship campus, we include instruction on structural racism and health disparities. Local experts explain the health care challenges as well as the health care and cultural resources in each neighborhood.

### Goal #2: Build upon health systems science curricula in undergraduate medical education

There are many similar challenges between design and implementation of the more established undergraduate curricula in HSS and the GME HSS curriculum that we designed and implemented. Program directors strongly preferred learning experiences that did not interfere with scheduled didactic sessions, as they worried about interference with teaching time for basic and clinical sciences necessary to pass American Board of Medical Specialties examinations.

Likewise, residents disliked activities away from clinical learning sites. We began with a requirement for live leadership training because of our belief that group learning is best for this competency. However, residents were concerned about potential time away from clinical education experience and interference with studying. This is similar to reported medical student resistance to undergraduate Health Systems Science curricula because of concern about interference with United States Medical Licensing Exams (USMLE) preparation [10]. Some Resident Forum members were vocal about the great difficulty of attending a 2–3-h session each year because of having to rearrange clinical coverage. Therefore, the Resident Forum agreed to assist by reviewing on-line leadership modules provided by our institution and recommending those they found most useful from a resident perspective for inclusion as options in the Leadership competency section of our curriculum. This was an example of compromise between educator preference for live leadership training and acceptance that some leadership training accepted by the residents is better than no leadership training.

Hospital leaders preferred a less experiential, more didactic curriculum with particular emphasis on adherence to coding and documentation and other policies. Undergraduate medical school leaders were also more familiar with classroom rather than experiential approaches, as didactic approaches are more uniform and measurable.

We allowed programs to modify learning experiences if they presented their alternative as a “best practice’ at our Graduate Medical Education Committee meeting for approval, and if they opened the learning activity to residents/fellows from other disciplines. This has resulted in sporadic increases in inter-disciplinary learning, usually at the time of an external guest facilitated workshop. The idea of sharing common curricular experiences across disciplines was new to our program directors, but we felt that it was important to learn health systems science as a health system. This difficulty has also been documented in the literature in medical schools instituting their own HSS curricula [10].

The list of alternative learning opportunities and contact information is posted on our GME intranet site in the section devoted to the Health Systems Science Curriculum for all programs to view. Examples of alternatives include a group discussion of a system issue followed by quality improvement project rather than individual reflective essays; this was appealing to our surgical specialists who felt that reflection alone felt too passive. Our psychiatry program offered their existing activity to trainees from other disciplines as a Population Health experience; they watch the 2017 documentary “Knife Skills” and then have dinner at the local restaurant that hires ex-convicts and was the focus of the movie.

### Goal #3: Provide our graduates with relevant knowledge, skills, and attitudes that impact their practice

There was significant anxiety upon introduction of the curriculum about detracting from opportunities to acquire medical knowledge, and concern that each discipline had unique educational needs such that an institutional curriculum might be harmful [11]. The anxiety abated over time and the HSS curriculum was less frequently discussed at Resident Forum.

Results of our annual GME internal surveys of faculty and residents show gradual progression in reflection and assessment of knowledge, skills, and attitudes that constitute our target competencies (see supplementary materials).

Trainees were also surveyed annually 11 months after orientation and asked about whether they applied the knowledge gained from the orientation “See the City You Serve” tour in a patient care setting. In 2019 40% answered “yes”. Even residents and fellows who have lived in our city for many years commented that they had never visited these neighborhoods and found the experience very worthwhile.

### Reflection

A limitation of our process is that the HSS curriculum is part of ongoing improvement in our health system, and it is not possible to define the exact contribution of the curriculum as opposed to other internal or external influences on our clinical learning environment. It is difficult to measure the impact on competencies of a curriculum that was modified as needed for a variety of disciplines.

- The ultimate measure of graduate outcomes is what physicians do in practice. Many of our programs have incorporated competencies in leadership, stewardship, and population health into their program aims. Success at meeting program aims is measured with periodic program level alumni surveys. We will encourage our programs to include questions in their graduate surveys that measure the graduates’ perception of how much these learning experiences benefited their professional lives and how marketable these experiences were in securing their first job. In 2015 as part of our GME strategic plan we articulated institutional aims for graduate performance including: Routinely seek perspective of other disciplines and professions for complex cases, practice high-value care, contribute to health policy, and work effectively within teams to ensure patient safety is the top priority.

We have found the establishment of an institutional curriculum invaluable to increase visibility and use of resources from our Leadership Institute, Quality Institute, and GME professional development intranet toward meeting aims for graduate outcomes. We continuously improve this curriculum as an improvement plan overseen by our GMEC Annual Institutional Review Committee. For example, our Palliative Care team has recently added a communication workshop on shared end of life goals of care to the Leadership offerings; our Clinical Transformation Team is developing mini-rotations as part of our Population Health offerings to sites such as rehabilitation hospitals, home health, and other sites that many trainees never experience.

Program aims are shared with applicants during recruitment. We expect that the HSS curricular aims will increasingly serve as a marketing tool despite only 7% of U.S. Seniors endorsing “opportunity for training in systems-based practice” as an important aspect of choosing a GME training program in the 2019 National Residency Matching Program survey, increasing to 7.5% in the 2021 survey [12].

## Conclusions

Use of our Health Systems Science Curriculum has been sustained for over four years, and the curriculum continues to evolve in response to user feedback. The curriculum was designed and implemented over approximately 18 months with only the costs of educators’ time, meaning that no dedicated budget for physical resources (other than buses for our orientation tour) was required.

Expansion of assessments for impact on clinical practice of our graduates after they have completed GME training and are engaged in independent practice may lead to further changes in our curriculum to better meet our goals. We require program directors to survey their graduates to assess program and institutional aims. We will have more data available to analyze in the coming years.

We believe that our experiences are translatable to other teaching hospitals. Each institution will need to consider the perspectives of their own stakeholders and the best approach to change management to create or improve the process of building new physician workforce competencies. These competencies should support institutional and program aims for graduate performance, and the degree to which graduates endorse behaviors consistent with these aims is the ultimate GME outcome metric.

## Supplementary Information

**Additional file ****1****: Supplementary Table 1.** Legend: Survey of Faculty (sent to approx. 650 core faculty each year, n= response) the year prior and the first two years after implementation of the curriculum. 1/3 respondents were program directors’. Note that all program directors, but not all faculty, participate in the curriculum.Supplementary Table 2:Legend:Survey of Residents and Fellows (sent to approx. 880 trainees each year, n= response) the year prior and the first two years after implementation of the curriculum.

## Data Availability

All data generated and analyzed for this study are included in the publication or the supplemental tables.
