# Supplementary material for: Design and implementation of a health systems science curriculum at a large teaching hospital
Source: BMC Med Educ. 2022 Aug 25;22:643. doi: 10.1186/s12909-022-03706-y (PMC9404630; doi:10.1186/s12909-022-03706-y)
Supplement: Supplementary file 1 — Additional file 1: Supplementary Table 1. Legend: Survey of Faculty (sent to approx. 650 core faculty each year, n= response) the year prior and the first two years after implementation of the curriculum. 1/3 respondents were program directors’. Note that all program directors, but not all faculty, participate in the curriculum.Supplementary Table 2:Legend:Survey of Residents and Fellows (sent to approx. 880 trainees each year, n= response) the year prior and the first two years after implementation of the curriculum. [file 12909_2022_3706_MOESM1_ESM.docx]

**Supplementary Tables**

**Supplementary Table 1:**

**Legend: Survey of Faculty (sent to approx. 650 core faculty each year, n= response) the year prior and the first two years after implementation of the curriculum. 1/3 respondents were program directors’. Note that all program directors, but not all faculty, participate in the curriculum.**

| **Are you assessed or asked to reflect on:** | **2017**  **Baseline**  **“often or consistently or sometimes”**  **N= 158** | **2018**  **First Year of Curriculum**  **“often or consistently or sometimes”**  **N= 160** | **2019**  **Second Year of Curriculum**  **“often or sometimes”  “rarely” eliminated**  **“never” remained**  **N= 205** |
| --- | --- | --- | --- |
| **Issues of cost, resources in health care (Stewardship)** | 40.1%  (n=66) | 48.7%  (n= 77) | 55.7 %  (n=134) |
| **Transitions of care** | 36.5%  (n=58) | 50.9%  (n= 82) | (supervision of)  85.2%  (n=173) |
| **Managing conflict or change (Leadership)** | 34.4%  (n=55) | 53.2%  (n=85) | 68.4%  (n=139) |
| **Analyzing data for health disparities (Population health)** | 24.7%  (n=40) | 42.4% (n=57) | 39.5%  (n=81) |

**Supplementary Table 2:**

**Legend: Survey of Residents and Fellows (sent to approx. 880 trainees each year, n= response) the year prior and the first two years after implementation of the curriculum**

| **Are you assessed or asked to reflect on:** | **2017**  **Baseline**  **“often or consistently or sometimes”**  **N= 126** | **2018**  **First Year of Curriculum**  **“often or consistently or sometimes”**  **N= 209** | **2019**  **Second Year of Curriculum**  **“often or sometimes” “rarely” eliminated**  **“never” remained**  **N= 213** |
| --- | --- | --- | --- |
| **Issues of cost, resources in health care (Stewardship)** | 57.3%  (n= 72) | 61.9%  (n=128) | 65.1%  (n=138) |
| **Transitions of care** | 68.8%  (n=86) | 64.3%  (n=133) | 76.9%  (n=163) |
| **Managing conflict or change (Leadership)** | 56.0%  (n=70) | 52.9%  (n= 109) | 68.4%  (n=145) |
| **Analyzing data for health disparities (Population health)**  **(changed in 2019)** | 43.5%  (n=53) | 47.3%  (n=97) | “Have you looked for healthcare disparities in QI, Research, or Clinical Data Sets”  Yes 64.9%  (n=133) |
